# Testing the Effect of Bathymetric Data Reduction on the Shape of the Digital Bottom Model

**DOI:** 10.3390/s23125445

**Published:** 2023-06-08

**Authors:** Wiktor Mujta, Marta Wlodarczyk-Sielicka, Andrzej Stateczny

**Affiliations:** 1 Marine Technology Ltd., 81-521 Gdynia, Poland; 2 Faculty of Navigation, Maritime University of Szczecin, Waly Chrobrego 1-2, 70-500 Szczecin, Poland; 3 Faculty of Civil and Environmental Engineering, Gdansk University of Technology, Gabriela Narutowicza 11-12, 80-233 Gdansk, Poland

**Keywords:** bathymetry, hydrography, data reduction, big data applications, data processing, data visualization, bathymetric data, bottom model

## Abstract

Depth data and the digital bottom model created from it are very important in the inland and coastal water zones studies and research. The paper undertakes the subject of bathymetric data processing using reduction methods and examines the impact of data reduction according to the resulting representations of the bottom surface in the form of numerical bottom models. Data reduction is an approach that is meant to reduce the size of the input dataset to make it easier and more efficient for analysis, transmission, storage and similar. For the purposes of this article, test datasets were created by discretizing a selected polynomial function. The real dataset, which was used to verify the analyzes, was acquired using an interferometric echosounder mounted on a HydroDron-1 autonomous survey vessel. The data were collected in the ribbon of Lake Klodno, Zawory. Data reduction was conducted in two commercial programs. Three equal reduction parameters were adopted for each algorithm. The research part of the paper presents the results of the conducted analyzes of the reduced bathymetric datasets based on the visual comparison of numerical bottom models, isobaths, and statistical parameters. The article contains tabular results with statistics, as well as the spatial visualization of the studied fragments of numerical bottom models and isobaths. This research is being used in the course of work on an innovative project that aims to develop a prototype of a multi-dimensional and multi-temporal coastal zone monitoring system using autonomous, unmanned floating platforms at a single survey pass.

## 1. Introduction

Knowledge of the Earth’s topography, considering the relief of the terrain and the mutual location of objects and landmarks is essential for many scientific fields and human investments [1,2]. Topography is a fundamental physical feature of our planet and supports research into its physics and dynamics [3,4,5]. A complete study of our planet’s topography is an extremely challenging task, since almost three-quarters of the planet’s surface is covered by water, and the depth in some areas can reach several kilometers [6]. At present, modeling of the seafloor surface can be performed based on measuring the depth of a body of water, resulting in a corresponding bathymetric dataset [7,8,9,10].

Bathymetric data describe the spatial representation of seafloor relief, while enabling the determination of the course of the isobaths, the location of underwater objects, the depth distribution, and the statistical parameters of the studied water area [11,12,13]. Bathymetry is one of the key variables in understanding marine dynamics and bottom sediment movements [14]. Spatially referenced depth data are a component of many works related to science, education, economics, and politics [15]. Bathymetric data also support: (a) the creation of marine cartographic studies in the form of navigation charts, improving the detection of potential navigational hazards and contributing to marine safety; (b) the study of dynamically occurring shoreline changes, including coastal erosion, ocean level rise, and the effects of climate change; (c) the preparation of hydrodynamic models, providing an auxiliary part in the calculation and prediction of currents, tides, and flood hazards; and (d) the study of the assemblage of marine living organisms associated with the bottom (benthos) and the determination of their habitat, reproduction, and feeding sites [15,16,17].

Acquisition of bathymetric data is performed based on platforms or survey vessels with measuring equipment integrated onboard. There are several survey systems for acquiring the water depth data, while their operating principle, operating environment, and delivered measurement accuracy significantly differ [18]. Currently, the most widely used measurement system is a multibeam system, which functions on the principles of aquatic acoustics [19,20,21] due to the significant limitations of electromagnetic wave propagation in the water medium [22,23,24]. The basic device of the multibeam system is a multibeam echosounder (MBES), which allows for the measurement of the depth distribution of a body of water. It has been used in surveys requiring a wide range of bottom surface coverage, along with the ability to detect objects in the water’s depth. The device has mainly found applications in bathymetric measurements with high depth values, as well as in shallow-water and near-shore measurements [25,26]. For coastal areas, modern unmanned craft are increasingly being used.

The basis of multibeam echosounder operation is the ability to generate single acoustic pulses in the water medium, which are captured by the device’s receiver after reflection from the surface of the bottom or another object. The projector is pointed in the direction of the seabed, generating an acoustic pulse with a specifically determined frequency, usually in the range of 100–500 kHz [27]. The captured acoustic signal is then subjected to an electronic beamforming process that uses signal processing techniques [25]. The created beams are then segmented, resulting in a set of soundings from a single acoustic pulse [28]. Spatial values are determined by measuring the bidirectional transition of the acoustic signal, the angle of inclination of the acoustic beam, and a refraction correction derived from the knowledge of the speed of acoustic wave propagation in specific layers of the water medium. The bidirectional transition time of the acoustic wave is calculated using implemented algorithms. Finally, the values are recalculated based on the provided refraction data, relating to the vessel’s spatial orientation and its position relative to the adopted reference system in each time unit [25,26].

Preliminary investigations and understandings of the characteristics of the acquired bathymetric data can be conducted based on a digital terrain model (DTM). A DTM is a discrete representation of the terrain surface with an applied interpolation algorithm, which makes it possible to calculate the height of any point within the model range [29]. In the general case, the interpolation method used can take the values of a certain function $f\left(x_{1}\right),\;f\left(x_{2}\right),\ldots ,f\left(x_{n}\right)$, for points belonging to a given interval $\left[x_{1},\;x_{n}\right]$. These points are referred to as interpolation nodes, which are the basis for making approximate determinations of the values of points that are not nodes [30,31].

DTM visualization allows us to perform the analysis in a more intuitive and less time-consuming way for humans. The scope of these analyzes can consider the shape of the studied bottom surface, and support the modeling of biological, geomorphological, and hydrological processes [29]. DTM makes it possible to show the peculiarities of the studied area and extract the basic elements from it. It also makes it possible to describe and extract relief structures, along with determining their parameters, such as slope, exposure, or relative height [32]. The creation of a digital terrain model based on data from a multibeam system usually follows the following steps: (1) acquiring a three-dimensional bathymetric dataset; (2) performing pre-processing of the data (including: noise removal, filtering, and data reduction); (3) creating a DTM based on the processed dataset; and (4) analyzing the DTM [33]. For underwater zone modeling, another name for this is the numerical bottom model (NBM) or the digital bottom model (DBM).

The use of the available spatial data reduction methods is becoming increasingly common [33]. The last decades of research on the application of acoustic devices in the water have contributed to the significant development of marine measurement systems. As a result of data acquisition via a multibeam system, a huge spatial dataset can be obtained, often identified with the term big data [34]. A raw dataset with a significant number of depth points forms the basis for creating a high-resolution final product. However, the use of such a set may not be necessary nor advisable as there are many disadvantages associated with it. Large datasets can significantly increase the data processing time and the required disk capacity. In addition, some works do not need very thorough processing, so data reduction may be the best solution in terms of the efficiency of data creation [35,36]. In practice, this is very often used.

Data reduction is intended to extract the most relevant parts of the data, while also reducing the volume or complexity of the data. The input dataset should be transformed in such a way that the output dataset remains representative of the study population [37]. When reducing bathymetric data, it is extremely important to preserve the feature points that could affect the subject of the study or its relevance to the safety of navigation. Existing techniques for reducing bathymetric data that are used during processing use interpolated values in the form of a regular GRID rectangle with an assumed grid size [38,39]. Commercial software designed for hydrographic applications often use reduction methods based on interpolating data to a specific cell size, sometimes resulting in the loss of characteristics of the original set [33,40]. A common way of doing this in practice is to represent a number of points using a single, specified by mean value, value closest to the mean, modal value or median [41]. There are few research papers available in the scientific literature on bathymetric data reduction methods. Several of these studies have included reduction methods based on the clustering of selected subsets of data using Kohonen neural networks (TBDRed) [42,43] or single- or multi-criteria optimization using the Douglas–Peucker algorithm for generalization [44,45]. Both of these methods do not consider the data interpolation process so that the actual spatial position of depth points can be preserved. It has also been shown that these methods can be fused, thereby obtaining more evenly distributed points, while preserving the most relevant depths and greater control over the number of points in the resulting set [33].

This paper is focused on the analysis of the bathymetric datasets obtained through the reduction process in commercial programs. The reduction of bathymetric data was performed using tools specifically designed for this purpose. The tools work on similar principles and preserve the original position of depth points. As part of our project, a multi-dimensional and multi-temporal coastal zone monitoring system will be created using an autonomous unmanned vessel. Modern sensors will be used for data acquisition: for underwater data—MBES and side-scan sonar, while for the terrestrial part—laser scanner and metric camera, respectively. The proposed system, apart from the possibility of integrating data from various sensors, thereby creating a multi-dimensional and multi-temporal database about a given coastal zone, will allow visualizing these data in the form of a spherical spatial map. Attention should be paid to the problem of heterogeneity of these systems and the large amounts of data, which are significant research challenges. Therefore, the problem of bathymetric data reduction has been thoroughly investigated in this study.

## 2. Test Areas

The study was conducted using three-point datasets in a three-dimensional space. The datasets were generated artificially, obtaining discrete equivalents of the selected mathematical function. In creating the datasets, an effort was made to replicate structures found in nature as closely as possible. The obtained datasets were then used to create digital bottom models with different characteristics of bottom relief.

### 2.1. The Process of Creating Analyzed Test Datasets

The test areas were created based on a mathematical function that shows the behavior of randomness and smoothing undulation. A function that shows both properties simultaneously can be termed as noise. The presented approach was shown first by Inigo Quilez. Similar approaches that enable the creation of reliable and irregular representations of the terrain surfaces using noise have often been used in computer graphics and cinematography.

The test datasets were generated using a piecewise function, which was written in the form of a third-degree polynomial function:

(1) $$N\left(x,y\right)=a_{ij}+\left(b_{ij}-a_{ij}\right)S\left(x-i\right)+(c_{ij}-a_{ij})S\left(y-j\right)\;+(a_{ij}-b_{ij}-c_{ij}+d_{ij})S\left(x-i\right)S\left(y-j\right)$$

where i, j=⌊x,y⌋ is the integer coordinates of the vertex of the interval, $a_{ij},b_{ij},c_{ij},d_{ij}$ are the parameters corresponding to the function values at the vertices of the interval, and $S\left(v\right)=3v^{2}-2v^{3}$ is the smoothing function, where v is the random values for the set for an area of 100 square meters. To ensure the consistency of combining all intervals, the common values of the vertices of adjacent intervals are used:

(2) $$a_{i,j}=b_{i-1,j}=c_{i,j-1}=d_{i-1,j-1}$$

The indication of the value of each vertex of the interval is performed based on any method of generating pseudorandom numbers. It is important that the number generator returns sufficiently different values so that the numerical representation of the created surface appears random. An example would be:

(3) $$a_{i,j}=2\left\{uv\left(u+v\right)\right\}-1$$

(4) $$\left(u,v\right)=50\left\{\frac{\left(i,j\right)}{\pi }\right\}$$

where *u* and *v* are zone-dependent random values. 

Completing the generation of these test sets requires two more steps. The first involves considering the rotation of the vector $p_{xy}=\left[x,\;y\right]$ using the rotation matrix $M_{o}$ to avoid overlapping the domains of the functions. The value of the rotation angle in this case can be arbitrary. The second step, on the other hand, considers the use of transverse compression and vertical contraction of the function to introduce increasing undulations. The final form of the function, which considers the combination of all polynomial functions, is as follows:

(5) $$f\left(p_{xy}\right)=\underset{n}{\sum }\frac{1}{2^{n}}N\left(M_{o}{}^{k}2^{n}p_{xy}\right)$$

It is worth noting that during the process of generating points, the spatial data were shifted in two-dimensional space by given values to realize the randomness of the mutual position of the points. This procedure was performed using the Box–Muller transformation, based on which it is possible to generate random numbers with a normal distribution, and based only on two values of a variable with a uniform distribution.

### 2.2. Characteristics of the Generated Test Datasets

The three generated test datasets were used to create digital bottom models. The test datasets were unified in terms of the number of points. Each contains 1,000,000 depth points distributed over an area of 100 m^2^, at about 0.01 m relative to each other. The resulting digital bottom models are characterized by different levels of variation and slope of the bottom surface, resulting in three different relief variants.

**Figure 1:**
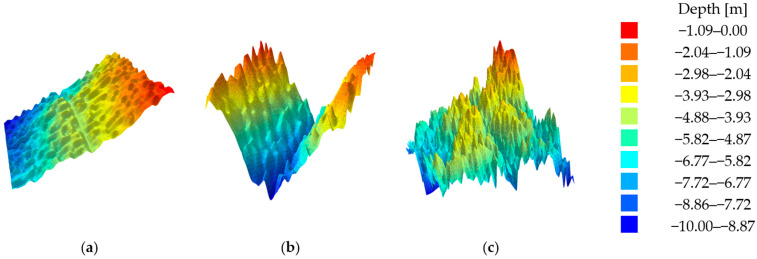
Visualization of the three test datasets using a digital terrain model in the form of a triangulated irregular network (TIN): (**a**) test dataset No. 1; (**b**) test dataset No. 2; and (**c**) test dataset No. 3.

A visualization of all three test datasets in the form of a digital terrain model is shown in Figure 1. The empirical distribution of the depth points and a summary of the basic statistics of the test datasets are shown in Table 1.

The first test dataset represents the low-relief seafloor surfaces with a global unidirectional slope. Disregarding the slope, the surface can be treated as flat bottoms, as there are no abrupt changes in the structure of the surface as a whole and no sudden local changes in the depth.

The second test dataset represents the seabed surfaces with a medium level of relief variation. The modelled bottom surface can be considered a fabricated riverbed. In terms of the entire surface, there were relatively larger local changes in the depth values than in the first test dataset.

The third test dataset represents the seabed surfaces with a high level of relief variation. The modelled seafloor surface can be considered extremely dangerous for navigation due to the occurrence of sudden changes in the depth. In terms of the entire surface, relatively the largest local changes in the depth values were observed compared to the first and second test datasets.

The range of depth in the test sets was chosen to increase the readability. The color palette is invariant for all test models throughout the document.

## 3. Methodology of the Research

Bathymetric data reductions were performed in two commercial programs. Geographic information system (GIS) software and software for hydrographic purposes were used. The first program was ArcGIS Pro, which is a modern GIS environment providing many professional tools for developing cartographic products in two-dimensional and three-dimensional space. This program enables the analysis, processing, visualization, management, and integration of data. The second software used was HYPACK, which includes a suite of programs designed for marine purposes. It consists of a wide range of tools necessary for the realization of bathymetric, sonar, and geophysical measurements.

The software provides bathymetric data reduction tools that preserve important depth points for navigation safety. The method used was reduce point density, which is implemented in ArcGIS software, and results in the reduction of bathymetric points in the input sets depending on the nominal start thinning radius parameter and the method of selecting individual points. The first parameter determines the radius of the circle within which a particular point will be selected, and the remaining points are thinned again. The second parameter was the selection method. Data reduction in the Hypack program was performed based on the sort module. This module is a kind of tool based on which it is possible to reduce the abundance of the bathymetric data, while guaranteeing the preservation of the minimum depths in the resulting set. The implemented algorithm searches for the smallest depth in a given section and reduces the points around the distance defined by the user as a parameter. These actions were repeated until a fully reduced dataset was obtained. The principle of operation of the implemented reduction algorithms inside these tools is similar. Both algorithms were designed to thin the input dataset according to a user-entered parameter of an approximate distance around a point. The most relevant points inside the circles of the radius chosen as the reduction distance parameter were selected, which preserves the original position of the depth points according to the input dataset, and thus does not interpolate the depth values of the points. The implementation of these algorithms ensure that point selection was not based on fixed distances between the points, and that the position of depth points in the resulting set is preserved relative to the original set.

**Figure 2:**
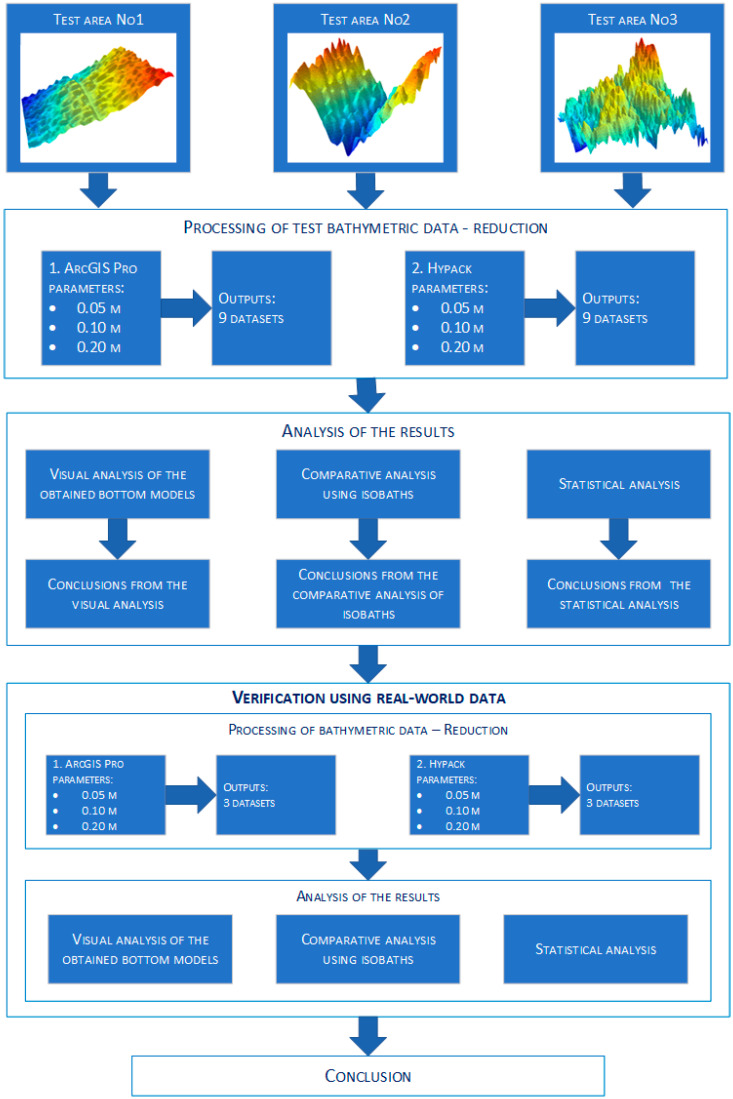
Diagram showing the research methodology.

The entire process of the research methodology is shown in the Figure 2 in the form of a diagram.

A comparative analysis was conducted between the raw bathymetric datasets and the resulting datasets obtained in the reduction process using the discussed algorithms and selected reduction parameters. The reduction parameters for each algorithm were taken as 0.05 m, 0.10 m, and 0.20 m, respectively. The degree of data reduction was determined according to the parameter of approximate distance—the radius of the circle. Such parameters were chosen after many trials during the study. The authors analyzed many possibilities. The selection of such parameters makes it possible to show the results satisfactorily. The authors cared about small values in particular, as the system being built will be used to create accurate bottom models. This system will be used in the coastal zone.

After reduction in two selected programs, a total of eighteen bottom models were obtained. The resulting models were subjected to extensive analysis consisting of three different components. The three various variants showed as many differences as possible between the considered datasets. The first involved a visual comparison that looks out for differences between the digital bottom models. The overall shape of the modeled bottom was be considered. The second variant used an approach incorporating isobaths and examines their course between the input sets. The last variant included a statistical comparison to examine for significant changes in the statistical parameters between the datasets. The number of points, dataset size, mean, standard deviation, median, range, minimum value, and maximum value were all taken into account during the analysis. After a comprehensive analysis, a periodic method was selected and then assessed on real data selected in a particular body of water. The water body and its characteristics were well known to the authors, so this method can be verified. In addition, a proprietary calibration cube was used during the verification stage to evaluate the selected reduction method.

## 4. Research and Results

### 4.1. Visual Analysis of Digital Bottom Models

**Figure 3:**
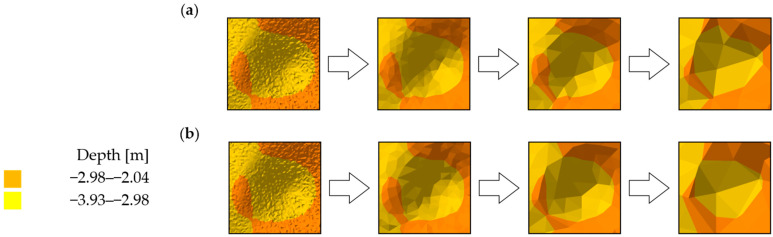
Selected fragments of the digital bottom model created from test dataset No. 1: (**a**) from the left: raw dataset; reduced dataset (ArcGIS Pro—reduction parameter 0.05 m); reduced dataset (ArcGIS Pro—reduction parameter 0.10 m); and reduced dataset (ArcGIS Pro—reduction parameter 0.20 m); (**b**) from the left: raw test dataset; reduced dataset (HYPACK—reduction parameter 0.05 m); reduced dataset (HYPACK—reduction parameter 0.10 m); and reduced dataset (HYPACK—reduction parameter 0.20 m).

**Figure 4:**
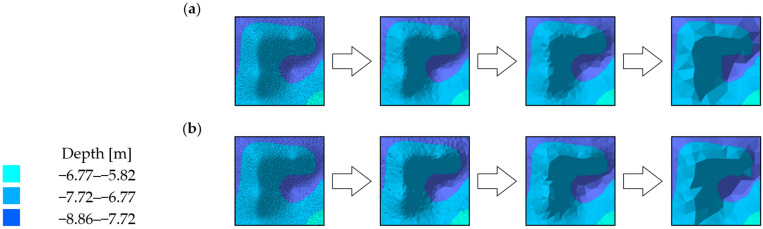
Selected fragments of the digital bottom model created from test dataset No. 2: (**a**) from the left: raw dataset; reduced dataset (ArcGIS Pro—reduction parameter 0.05 m); reduced dataset (ArcGIS Pro—reduction parameter 0.10 m); and reduced dataset (ArcGIS Pro—reduction parameter 0.20 m); (**b**) from the left: raw test dataset; reduced dataset (HYPACK—reduction parameter 0.05 m); reduced dataset (HYPACK—reduction parameter 0.10 m); and reduced dataset (HYPACK—reduction parameter 0.20 m).

**Figure 5:**
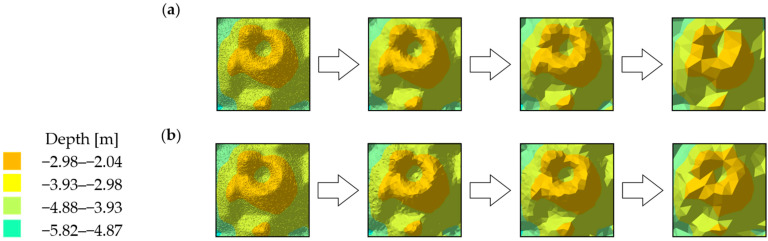
Selected fragments of the digital bottom model created from test dataset No. 3: (**a**) from the left: raw dataset; reduced dataset (ArcGIS Pro—reduction parameter 0.05 m); reduced dataset (ArcGIS Pro—reduction parameter 0.10 m); and reduced dataset (ArcGIS Pro—reduction parameter 0.20 m); (**b**) from the left: raw test dataset; reduced dataset (HYPACK—reduction parameter 0.05 m); reduced dataset (HYPACK—reduction parameter 0.10 m); and reduced dataset (HYPACK—reduction parameter 0.20 m).

The visual comparative analysis was conducted based on digital bottom models, which were generated from the raw and reduced test datasets. The numerical bottom models were juxtaposed against each other based on the individual test area and the reduction parameter, thus enabling the comparison of the raw datasets against the reduced datasets. Selected sections of the numerical bottom models, showing changes in their shape depending on the reduction parameter used, and the degree of variation in their bottom topography, are shown in Figure 3, Figure 4 and Figure 5.

Test surfaces obtained from reduced test dataset 1 with different reduction parameters and algorithms were found to have affected the visual shape change (Figure 3). Fragments of the model obtained from the set with the smallest degree of reduction least affected the shape change of the surface. As the reduction parameter increased, the degree of simplification of the model surface also increased. Simplification of the model caused little change in the shape of the areas with locally similar depth values. The change in the shape of areas formed by the set values of the depth points between the algorithms remained similar. The depth points retained their actual positions relative to the original set. The algorithms, despite searching for the smallest depth values, selected different points. No digital terrain model revealed significant differences in the depth points of the key areas between the original and reduced sets.

Test surfaces with a medium degree of relief variation obtained from test dataset No. 2 showed further changes in the shape of the model surface (Figure 4). The increase in relief variation was found to have affected the preservation of greater shape fidelity of the shallower areas. The priority of selecting the shallowest points and the reduced significance of selecting the deepest points were also observed. The number of points in the deeper areas was reduced more significantly, thereby preserving a greater fidelity of the shallower regions of the model. The more the sudden and relatively large fluctuations in the depth values observed, the deeper the areas were simplified in favor of preserving the shallower areas. Digital bottom models obtained from the reduced sets again showed fidelity in the shape and preservation of the feature points relative to the original set.

The test surfaces with the highest degree of relief variation showed a high shape fidelity to the original surface (Figure 5). A slight simplification of the geometry of areas of similar depths was visible, resulting from a reduction in the number of vertices of the triangulated irregular network. Similar to the comparison of test surface fragments No. 1 and 2, as the value of the reduction parameter increased, there was a gradual loss of information on the deeper areas, which are surrounded by shallower depth points. Also noticeable was an improvement in the visual shape of some areas of the model, which was created from the resulting set from the HYPACK program. This may be a result of the visible increase in the number of points building the numerical bottom model. All the obtained digital models demonstrated a high fidelity to the original model.

The reduction of bathymetric datasets affected the visual simplification of the models and noticeable changes in the shape of the bottom surface. The reduction primarily affected the density distribution of the depth points in that some areas in the surface models differed in shape from the reference model. Visually, the similarity of the mechanisms of the functioning of the algorithms in ArcGIS Pro and HYPACK software was demonstrated. As intended, the applied bathymetric data reduction algorithms showed the priority of preserving the shallowest points according to the input dataset, thereby prioritizing the safety of marine navigation. In addition, the use of different reduction parameters caused changes in the shape of the considered digital bottom models. The higher the value of the reduction parameter was adopted, the greater the simplification of the output model was obtained. All numerical bottom models showed a high fidelity of relief to the reference models.

### 4.2. Comparative Analysis Using Isobaths

**Figure 6:**
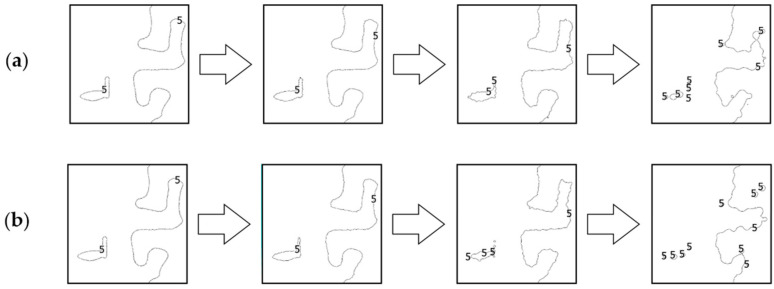
Selected fragments of the isobaths created from test dataset No. 1: (**a**) from the left: raw dataset; reduced dataset (ArcGIS Pro—reduction parameter 0.05 m); reduced dataset (ArcGIS Pro—reduction parameter 0.10 m); and reduced dataset (ArcGIS Pro—reduction parameter 0.20 m); (**b**) from the left: raw test dataset; reduced dataset (HYPACK—reduction parameter 0.05 m); reduced dataset (HYPACK—reduction parameter 0.10 m); and reduced dataset (HYPACK—reduction parameter 0.20 m).

**Figure 7:**
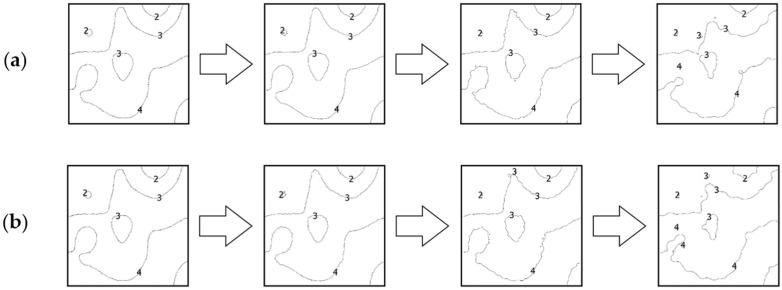
Selected fragments of the isobaths created from test dataset No. 2: (**a**) from the left: raw dataset; reduced dataset (ArcGIS Pro—reduction parameter 0.05 m); reduced dataset (ArcGIS Pro—reduction parameter 0.10 m); and reduced dataset (ArcGIS Pro—reduction parameter 0.20 m); (**b**) from the left: raw test dataset; reduced dataset (HYPACK—reduction parameter 0.05 m); reduced dataset (HYPACK—reduction parameter 0.10 m); and reduced dataset (HYPACK—reduction parameter 0.20 m).

**Figure 8:**
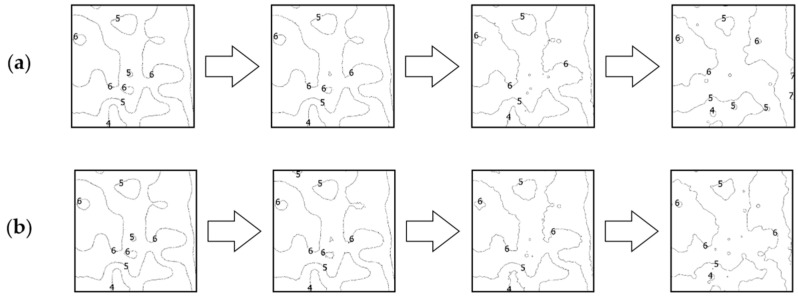
Selected fragments of the isobaths created from test dataset No. 3: (**a**) from the left: raw dataset; reduced dataset (ArcGIS Pro—reduction parameter 0.05 m); reduced dataset (ArcGIS Pro—reduction parameter 0.10 m); and reduced dataset (ArcGIS Pro—reduction parameter 0.20 m); (**b**) from the left: raw test dataset; reduced dataset (HYPACK—reduction parameter 0.05 m); reduced dataset (HYPACK—reduction parameter 0.10 m); and reduced dataset (HYPACK—reduction parameter 0.20 m).

The second analysis was performed by comparing the shape of isobaths, which are defined as curves connecting points with the same depths. The isobaths were formed from the raw and reduced test datasets. The interval of the isobaths was set as 1 m. Selected fragments of isobaths, showing changes in their shape depending on the reduction parameter used and the degree of variation in bottom topography are shown in Figure 6, Figure 7 and Figure 8.

All fragments of the isobaths that were created from reduced test dataset No. 1 showed a change in their shape. The isobaths obtained from the reduced dataset with a reduction parameter of 0.05 m showed no significant differences between the shape of the reference isobaths. As the reduction parameter increased, the shape of the isobaths became increasingly distorted as a result. The isobaths obtained from the reduced sets with a reduction parameter of 0.10 m differed in their shape from the reference isobaths. In the case of the reduction parameter of 0.20 m, there was a partial loss of information regarding the original course of the isobaths. Some parts of the isobaths were also split into minor curves.

The shape of the Isobaths obtained from reduced test dataset No. 2 was also found to have changed. Reduction of the test set with the lowest parameter slightly affected the shape of the isobaths. Despite the formation of minor curves, no major changes were shown with respect to the reference isobaths. An increase in the reduction parameter to a value of 0.10 m resulted in visible simplifications in some parts of the isobaths. More intermediate isobaths were created, however, the overall shape of the isobaths remained preserved. The isobaths obtained from the most reduced datasets showed the greatest change and difference from their original course. However, it was possible to indicate the corresponding parts in comparison with the original isobaths.

The isobaths obtained from reduced test dataset No. 3, characterized by the highest degree of relief variation in this study, showed the smallest shape changes compared to the other two test datasets. The reduction parameter of 0.05 m did not significantly affect the change in the shape of the isobaths. Only a slight simplification of the geometry of the curves was observed, but with the preservation of the most important information for navigation safety. In the case of isobaths created from a dataset with a reduction parameter of 0.10 m, the fidelity of the isobaths to the reference isobaths was preserved in shallower areas. Some of the information regarding the deepest points was lost in favor of preserving the shallower points. Using a reduction parameter of 0.20 m, shape deformations relative to the reference isobaths were shown. Only the general shape of the isobaths for the shallowest areas was preserved, splitting adjacent isobaths into their minor equivalents.

The effect of the reduction of bathymetric data on the course and shape of the isobaths analyzed was made apparent. The reduction methods showed similarities, which were determined by the similar course of isobaths for the corresponding reduction parameters. An increase in the value of the reduction parameter exhibited the greatest effect on changing the course of isobaths at the deepest points. The shape of the isobaths at the deepest points was deformed to a degree corresponding to the applied value of the reduction parameter. The isobaths at the shallowest points showed the greatest fidelity of both the shape and course relative to the original isobaths. The overall shape of the isobaths in the shallowest areas was preserved, regardless of the values of the reduction parameter used. As the reduction parameter increased, the number of minor adjacent isobaths that derived from the original course of the curves also increased as a result.

### 4.3. Comparative Analysis Using Statistical Parameters

The third analysis involved a comparison of the basic statistical parameters, determined from the raw and reduced bathymetric datasets. Statistical measures of the test datasets with different degrees of reduction were summarized according to the reduction parameters used. The statistical measures are shown in Table 2, Table 3 and Table 4.

The number of depth points in the reduced datasets was significantly reduced, resulting in a reduction in the size of the datasets by about 94–99%. The effect of the reduction parameters on the degree of the reduction of the number of depth points was shown. Regardless of the algorithm used, corresponding datasets with the same reduction parameters maintained the number of points at a similar level. The size of the sets between the algorithms varied within 1%. It should be emphasized that a very large amount of data was reduced, and yet the shape of the bottom was not substantially deformed and corresponded to the final requirements.

The mean value of depth in the reduced datasets was preserved relative to the non-reduced dataset, causing only differences of 0.01–0.02 m. The measure of the dispersion of the depth values relative to their mean remained nearly unchanged, showing no significant deviations between the datasets.

The median value of the resulting datasets differed between the reduction algorithms used. In the ArcGIS Pro datasets, the median value remained nearly unchanged, however the HYPACK datasets revealed changes in its value as the reduction parameter increased.

The shallowest points in the reduced datasets were preserved relative to the raw datasets. The priority of selecting the points of the greatest importance for navigational safety in the algorithms used was therefore confirmed. For the deepest points, there were differences between the reduction parameters used. The resulting datasets from both programs considered almost the same depth values, while as the reduction parameter increased, the values started to differ from the original value.

The number of depth points was visibly reduced between the bathymetric datasets. The size of the test dataset was reduced by about 92–99%, depending on the reduction parameter used. The resulting datasets from the ArcGIS Pro software preserved a relatively similar number of points as the datasets with the corresponding reduction parameters presented in the analysis of test dataset No. 1. In contrast to ArcGIS Pro, the resulting datasets obtained from the HYPACK software showed a change in the number of points preserved compared to test dataset No. 1. The number of points in the resulting dataset increased, from which it can be initially assumed that as the variation and complexity of the relief increases, the algorithm implemented in HYPACK can retain a greater number of depth points as a result.

The mean depth value increased with the increasing simplification of the test dataset in all the reduced bathymetric datasets. This was due to the need to store the shallowest points so that the deeper points were thinned out the most. The greatest difference in values was obtained in the result set from ArcGIS Pro, with the highest reduction parameter used. The datasets reduced in HYPACK demonstrated a higher fidelity, receiving the greatest value differences of 0.03 m. This may have been influenced by the preservation of more points in the dataset obtained from HYPACK. The standard deviation remained almost unchanged between the datasets, showing differences of 0.01–0.02 m.

Depending on the algorithm used, the median values differed between the reduced sets. The median value in the datasets obtained from ArcGIS Pro increased as the reduction parameter increased. The maximum value difference was 0.08 m. For the result datasets from HYPACK, the median value initially decreased, reaching a maximum value difference of 0.06 m, and then started to increase as the value of the reduction parameter increased.

The reduced test datasets preserved the shallowest points from the raw dataset. The selection of the deepest points varied depending on the reduction algorithm used. In the case of ArcGIS Pro, the largest value difference between the raw and the most reduced dataset was 0.02 m, while in the dataset from HYPACK the difference was 0.05 m, respectively.

The number of depth points was reduced in all reduced datasets. The size of these datasets was reduced by about 88–99%, relative to the raw test dataset. The number of points in the resulting dataset from ArcGIS Pro was similar to the shown number of points in the previous test datasets. In the case of the reduced datasets in HYPACK, the selected number of points increased again, thereby preserving a higher number of points than in the reduced test dataset No. 2. The high variation in relief influenced an almost three-fold increase in the number of depth points preserved, compared to the datasets with a low variation in their surface area.

The mean value of depth in the reduced datasets increased as the value of the parameter used for reduction also increased. The mean values in the resulting datasets from both programs did not differ significantly from the mean value of the non-reduced dataset. The maximum value difference between the most reduced and raw dataset for ArcGIS Pro was 0.08 m, while for HYPACK it was 0.05 m, respectively. The standard deviation again showed no significant change, obtaining the largest value difference of 0.02 m.

The median value In all cases increased as the value of the reduction parameter correspondingly increased. The largest difference in the median value between the reduced and raw dataset for ArcGIS Pro was 0.10 m, while for HYPACK it was 0.09 m, respectively.

As noted in the analyzes of previous datasets, the shallowest points from the raw dataset were preserved in all the reduced datasets. The values of the deepest points differed between the reduction algorithms used. The datasets reduced in ArcGIS Pro showed the greatest difference of 0.15 m compared to the raw dataset. With HYPACK, the resulting difference was noticeably smaller, resulting in a 0.06 m difference. This could have been influenced by the significantly higher number of points preserved in the resulting dataset.

The number of depth points in all datasets decreased as the reduction parameter increased. The reduced datasets in ArcGIS Pro showed a similar number of depth points between the test datasets using the same reduction parameters. No significant effect of surface variation on increasing the number of selected points was observed. In the case of the result sets from HYPACK, a significant change in the number of selected points was shown, which was dependent on the degree of surface variation created by the input dataset. As the diversity of the depth points increased, the number of points in the reduced datasets also increased accordingly to preserve the shape and course of the surface as closely as possible.

The mean values of the reduced datasets, obtained from the least varied input dataset, did not reveal any significant changes between each other. Only as the variation of the depth points in the input set increased did the resulting sets start to differ significantly from the original mean value. The more differentiated and reduced the dataset, the greater the differences that were obtained in relation to the raw set. The smallest differences were obtained based on the result datasets from HYPACK. This was formed as a result of keeping the greatest number of depth points in the result datasets between the programs used. The standard deviation revealed no significant differences in any variant. The values were relatively similar, regardless of the variation of the depth points in the input dataset and the reduction parameter used.

The median values also showed no significant differences between the raw dataset with the least variation and the corresponding reduced datasets. As the variation of the datasets increased, greater differences in the median values between them started to show. The resulting datasets from both programs showed similar difference values for the same dataset variation and the reduction parameter used.

All reduced bathymetric datasets preserved the shallowest points in accordance with the algorithms’ assumptions for selecting the points of the greatest importance for navigational safety. The deepest points in the result sets were selected depending on the algorithm and the reduction parameter used, as well as the variation of the test data. The algorithms used in both programs preserved similar deepest points, revealing no significant differences between them.

## 5. Verification of Results on Real Bathymetric Data

Verification of the analyzes was conducted using the real bathymetric dataset. Depth point reduction was performed using the bathymetric data reduction algorithm, which was implemented as a tool in ArcGIS Pro. Our additional goal was to develop our own method of reduction and compare it with the one chosen here.

**Figure 9:**
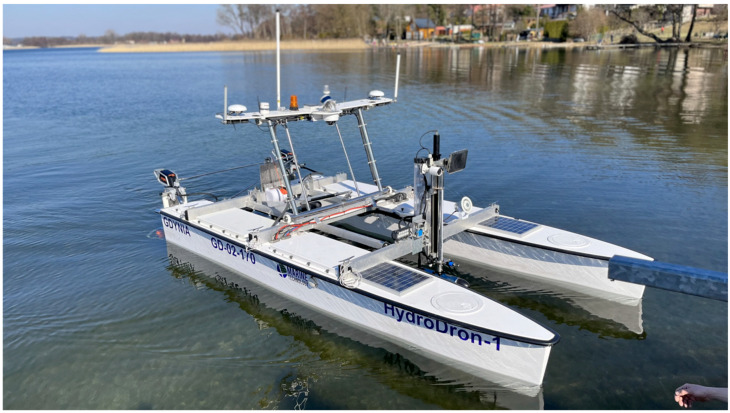
Autonomous survey vessel—HydroDron-1.

The actual bathymetric dataset was acquired using the PING DSP 3DSS-DX-450 interferometric echosounder mounted on the HydroDron-1 autonomous survey vessel (Figure 9). HydroDron-1 is a double-hulled catamaran-type vessel with a draft of approximately 0.20 m at the bow and approximately 0.50 m at the stern. It is equipped with several peripherals, measuring devices, and navigation sensors, which are integrated with each other through two industrial computers.

Communication with the autonomous vessel takes place through a radio mast, which emits and receives electromagnetic waves in a pre-defined frequency band, determined according to the surrounding interference. All data is transmitted to the shore station, which consists of a control console and a navigation console, on which the modified Mission Planner program is installed. An additional operating computer permits connection to a remote desktop computer located on the vessel, so that the recording of the bathymetric data can be managed.

**Figure 10:**
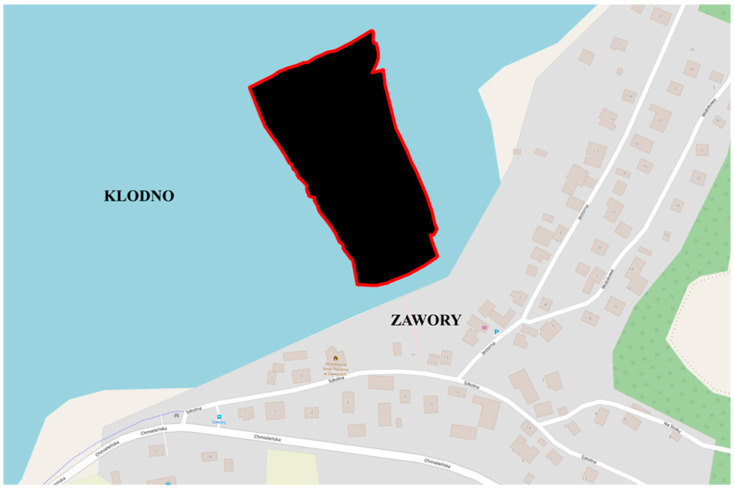
The test area located on Klodno Lake.

Bathymetric data acquisition was conducted as part of the 4DShoreMap project, which includes an innovative system for the multi-dimensional and multi-temporal monitoring of the coastal zone using an autonomous vessel. Currently, Klodno Lake is not covered by shore zone monitoring. However, it is one of the project’s test areas. The dataset was acquired on Lake Klodno, which is a ribbon lake with a maximum depth of 38.5 m (Figure 10). The raw dataset consisted of 13,614,483 depth points. The area surveyed comprised a flat bottom with low relief variation. The shallowest point was at a depth of 1.83 m, while the deepest point was at a depth of 16.77 m, respectively.

**Figure 11:**
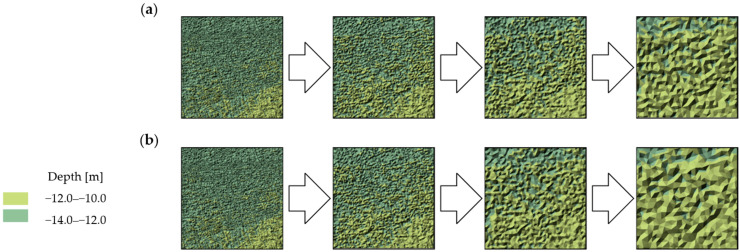
Selected fragment of the digital bottom model No. 1 created from the test dataset: (**a**) from the left: raw dataset; reduced dataset (ArcGIS Pro—reduction parameter 0.05 m); reduced dataset (ArcGIS Pro—reduction parameter 0.10 m); and reduced dataset (ArcGIS Pro—reduction parameter 0.20 m); (**b**) from the left: raw test dataset; reduced dataset (HYPACK—reduction parameter 0.05 m); reduced dataset (HYPACK—reduction parameter 0.10 m); and reduced dataset (HYPACK—reduction parameter 0.20 m).

A numerical terrain model was created from the acquired bathymetric data. The numerical model did not show any major objects on the bottom surface, apart from a specially placed cube that was used to calibrate the survey equipment. The dataset was then subjected to reduction. During the reduction of the bathymetric data, a reduction parameter of 0.05 n, 0.10 m, and 0.20 m were adopted to show an intermediate degree of surface simplification. Numerical bottom models obtained from the reduced sets have been presented in Figure 11.

The surveyed area covered 25 square meters. On the raw dataset, a gentle slope can be seen, which has a changing depth from about 11 m to 13 m. It can be seen that the raw dataset is very densely distributed. When the reduction parameter of 0.05 m was applied, the data obtained with ArcGIS software was found to be very similar to the data obtained with Hypack software. The differences in the shape of the surface were small. In the case of the parameter at the level of 0.10 m, we imminently observed larger differences. Both surfaces were slightly shallow—more shallower areas were observed, which was deemed to be related to the operation of the reduction methods. The Hypack software was found to have reduced the input dataset more, containing fewer node points visible on the output model. The last largest parameter was found to have reduced the studied area the most. In this case, the observed differences were the largest. With the use of Hypack software, the surface changed much more significantly. The surface that most resembles the model surface was the one obtained with ArcGIS software with a parameter of 0.05 m and 0.10 m, and the surface obtained with Hypack software with a parameter of 0.05 m, respectively.

**Figure 12:**
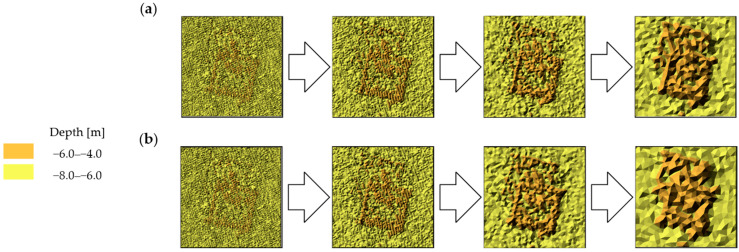
Selected fragment of the digital bottom model No. 2 created from the test dataset: (**a**) from the left: raw dataset; reduced dataset (ArcGIS Pro—reduction parameter 0.05 m); reduced dataset (ArcGIS Pro—reduction parameter 0.10 m); and reduced dataset (ArcGIS Pro—reduction parameter 0.20 m); (**b**) from the left: raw test dataset; reduced dataset (HYPACK—reduction parameter 0.05 m); reduced dataset (HYPACK—reduction parameter 0.10 m); and reduced dataset (HYPACK—reduction parameter 0.20 m).

In the next step, the authors selected another test area for comparison. This area also covered 25 square meters and contained the object lying on the bottom. The areas obtained with the source data and the reduced data for this area are shown in Figure 12.

In the model obtained from the raw data, an object lying on the bottom was observed. This object was about two meters high and shaped similar to a square. This was a calibration cube laid down prior to the measurements—for research purposes. The surfaces obtained from the reduced data gave very similar results to the previously analyzed surfaces. In the case of the reduction parameter of 0.05 m, both programs obtained similar results. Moreover, the outline of the underlying object at multiple nodal points was still observed. The reduced surface obtained with ArcGIS with a parameter of 0.10 m still shows a clearly lying bottom object. In other cases, we still observe a significant upwelling, but already, the shape of the object has changed significantly.

**Figure 13:**
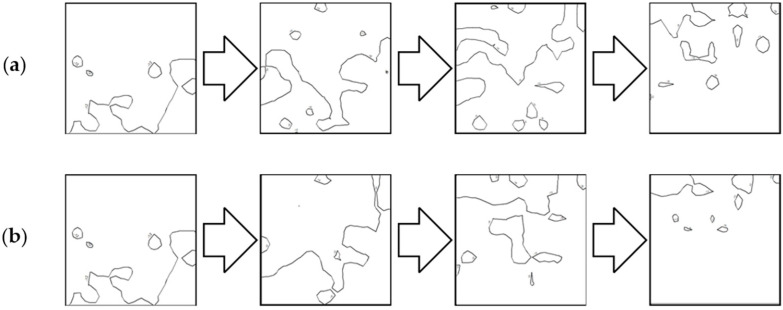
Selected fragments of the No.1 isobaths created from the test dataset: (**a**) from the left: raw dataset; reduced dataset (ArcGIS Pro—reduction parameter 0.05 m); reduced dataset (ArcGIS Pro—reduction parameter 0.10 m); and reduced dataset (ArcGIS Pro—reduction parameter 0.20 m); (**b**) from the left: raw test dataset; reduced dataset (HYPACK—reduction parameter 0.05 m); reduced dataset (HYPACK—reduction parameter 0.10 m); and reduced dataset (HYPACK—reduction parameter 0.20 m).

**Figure 14:**
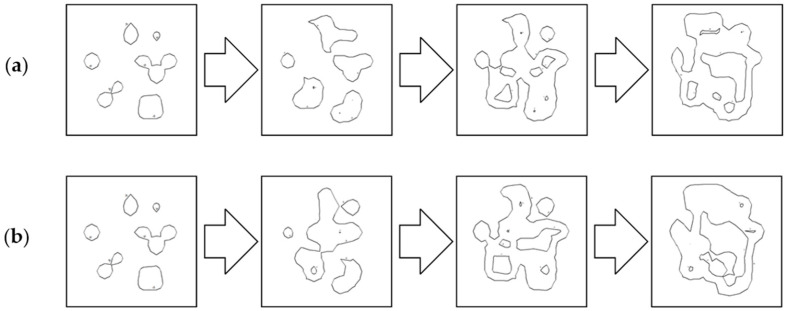
Selected fragments of the No.2 isobaths created from the test dataset: (**a**) from the left: raw dataset; reduced dataset (ArcGIS Pro—reduction parameter 0.05 m); reduced dataset (ArcGIS Pro—reduction parameter 0.10 m); and reduced dataset (ArcGIS Pro—reduction parameter 0.20 m); (**b**) from left: raw test dataset; reduced dataset (HYPACK—reduction parameter 0.05 m); reduced dataset (HYPACK—reduction parameter 0.10 m); and reduced dataset (HYPACK—reduction parameter 0.20 m).

In the next step, the authors represented the real areas analyzed above using isobaths. The interval for isobaths was set at 1 m. Selected fragments of isobaths, showing changes in their shape depending on the reduction parameter used are shown in Figure 13 and Figure 14.

As with the visualization of the surface in the form of 3D models, the isobaths provided similar results. The last parameter at 0.20 m completely changed the isobaths. The same was true for the data obtained with the Hypack software with a parameter of 0.10 m. The area was significantly shallowed, and the course of the isobaths was completely different. The best results were obtained for the ArcGIS software with a parameter of 0.05 m and 0.10 m, and with the Hypack software with a parameter of 0.05 m, respectively, and we observed the similarity for the resulting isobaths. It should be mentioned that the performance of both programs was deemed to be related to the fact that the depths of the smaller ones were more relevant during the reduction. Another area shown using isobaths was the area encompassing the calibration cube lying on the bottom (as shown in Figure 14).

In this visual analysis, the authors focused on knowing the shape of the underlying object, which had a cube shape. The isobaths obtained from the source data did not show this shape as they were very densely distributed, and the isobaths obtained from them had values at the limit of the depth of the lying object. However, it should be remembered that this analysis also aimed to be utilized for comparison with the source isobaths. Here, considering both of these factors, i.e., the known shape of the object and the source isobaths, it can be concluded that the best results were obtained for the data reduced with the ArcGIS software with the parameter set to 0.10 m. The shallows have not changed shape, and the object lying on the bottom was clearly visible.

In the next step, the authors calculated statistical values for all the depth points after reduction with the given parameters. The statistical measures are shown in Table 5.

The source dataset contained 13,614,483 depth points. Visualization of these points and their subsequent analysis was very time-consuming due to the large size of the collection. After reduction with the two software packages, the collection decreased significantly. In the case of the ArcGIS program, for the parameter of 0.05 m the dataset decreased by 75.40%, for the parameter of 0.10 m the dataset decreased by 87.13%, while for the parameter of 0.20 m the dataset decreased by 96.25%, respectively. It should be clear that the Hypack software reduced the input set more—the resulting output sets were smaller than in the case of ArcGIS software. The average depth of the source set was 9.66 m. The closest results were obtained for ArcGIS software with parameters of 0.05 m and 0.10 m–9.99 m, respectively. The minimum depth values were the same for all instances—1.83 m. This was deemed to be related to the significance of the shallower areas in the studied water bodies. The maximum depths for the studied result sets decreased as the reduction parameters increased, as did the value of the standard deviation.

When summarizing the results obtained, it is important to consider all the results: those obtained with the test data and with the real data. In addition to the analysis of the statistical results, previous visual analyzes of the surface and isobaths should also be taken into account. Considering the visual evaluation of the numerical bottom model for the simulated data, the similarity of the mechanism of operation of the algorithms in ArcGIS Pro and HYPACK software was demonstrated. The application of different reduction parameters resulted in changes in the shape of the considered digital bottom models. For test area No. 2 and test area No. 3, the best results were obtained with ArcGIS software and Hypack software for parameters 0.05 and 0.10, respectively (Figure 4 and Figure 5). However, for test area No. 1, the best results were obtained for the same parameters as with the real data (Figure 3). For the numerical bottom model, during the analysis of the selected areas, it was found that the best results were obtained with the ArcGIS software with a parameter of 0.05 m and 0.10 m, and with the Hypack software with a parameter of 0.05 m, respectively. Isobaths generated during reduction also yielded similarities, with the results obtained being similar. The increase in the value of the reduction parameter had the greatest effect on changing the course of the isobaths at the deepest points. The shape of the isobaths at the deepest points underwent the greatest deformation. The overall shape of the isobaths in the shallowest areas was preserved. Considering all the analyzed surfaces (the simulated ones and the real ones), the best results were obtained with the ArcGIS software with a parameter of 0.05 m and 0.10 m, and with the Hypack software with a parameter of 0.05 m, respectively. However, analyzing the isobaths obtained from the real data where we see an object lying on the bottom, it should be concluded that the best results were obtained with the ArcGIS program with a parameter of 0.10 m. Considering the statistics in the case of very high-density test data, the algorithms used in both programs preserved similar deepest points, showing no significant differences between them. The same can be said for results obtained from real data. However, the data reduced with Hypack software had fewer depth points—the datasets have been reduced more. As intended, the bathymetric data reduction algorithms used showed priority for preserving the shallowest points according to the input dataset. It should be noted that different bottom surface shapes and different distributions of bathymetric points were used during the study. The theoretical data that were computer-generated had a very different shape. In contrast, the actual data collected on Klodno Lake had a gentle slope and an object lying on the bottom. The results that the authors obtained using the real data thus confirm the research on the simulated data.

In summary, the best results were obtained for the reduction with the ArcGIS program for the 0.10 m parameter. Therefore, in the next section, the authors decided to show detailed results for these settings—statistics and a numerical bottom model of the entire study area. Table 6 shows the summary of the statistical measures of the raw and reduced test datasets in ArcGIS software with a reduction parameter of 0.10 m.

The dataset was reduced by 11,862,145 bathymetric points, accounting for 87.13% of the total collection. The mean depth of the points after the reduction was −9.99 m, and the maximum depth decreased by 6 cm, while the minimum depth in the study area remained the same. Such a reduced bathymetric dataset allows for the creation of an accurate bottom model with minimum depth. The speed of the calculations and the speed of the overall analysis on such a reduced set increases significantly as a result. Data export before reduction to the project geodatabase was 12 min. In contrast, the export of the already reduced dataset was only 1 min. This makes it significantly easier to work with such large datasets. A reduced dataset reduces the storage capacity required and reduces the required data transfer time over a specific network. It further facilitates the visualization of such a dataset and its understanding.

**Figure 15:**
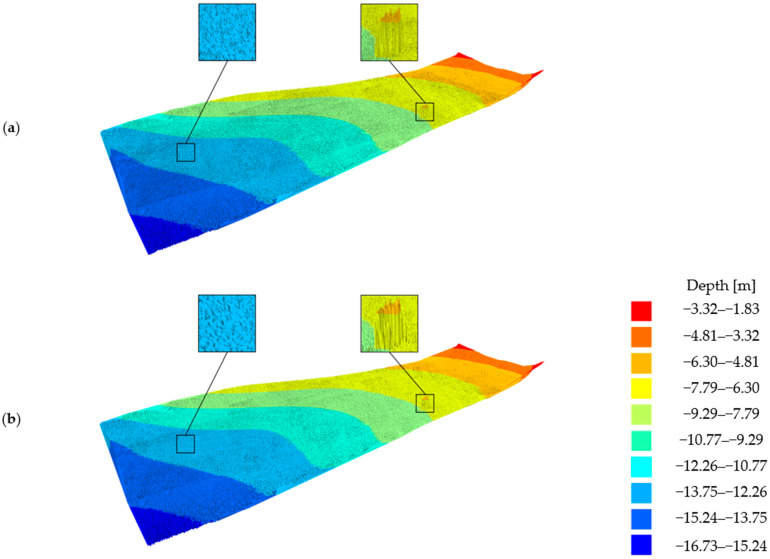
Visualization of the digital bottom model created from the real bathymetric dataset: (**a**) raw bathymetric dataset; and (**b**) reduced bathymetric dataset in ArcGIS Pro software with a reduction parameter of 0.10 m.

A numerical terrain model was once again created representing the reduced dataset. The model revealed no significant differences from the original model. The shallowest points were preserved according to the non-reduced dataset. Areas of the model with similar depths were simplified but kept the fidelity of the original surface. The numerical bottom models are shown in Figure 15.

The numerical bottom model, which was created from a reduced real dataset, shows no significant differences between the original model. Areas of the model with similar depth values were preserved. The shape and structure of the modeled surface have been simplified slightly, however, without showing the loss of the most relevant information for navigation safety.

## 6. Conclusions

The reduction of the bathymetric datasets resulted in a decrease in the number of depth points in all the output sets, relative to the number of points in the original set. The number of points in the resulting datasets decreased as the value of the reduction parameter increased. The reduction in the number of depth points in the datasets resulted in a visual simplification of the digital bottom models, along with visible changes in the topography of the bottom surface. A change in the density of the distribution of the points on the bottom surface caused a partial discrepancy in the shape of several zones of the resulting models, depending on the algorithm and the reduction parameter used. Applying an increasing value of the reduction parameter led to an increased degree of change in the shape of the bottom surface relative to the reference model.

The original course of the isobaths was preserved at locally shallower points, regardless of the reduction parameter used in the algorithm. The curves connecting the deeper points revealed several inaccuracies in comparison to the reference isobaths, thus losing information. The greater the reduction parameter used, the more the isobaths differed from the original course. Increasing the value of the reduction parameter also resulted in the elimination of isobaths in relatively deeper areas and visibly simplified the shape of the curves.

Statistical measures revealed no significant differences between the datasets with a low variation in the depth points. An increase in the variation of the bottom area, on the other hand, resulted in noticeable changes in the received values. The more frequent and greater the variations in depth occurred, the greater the differences in measures that were noted between the reduced and raw datasets. The most susceptible to change in the variation of the input dataset were the mean, median, and greatest depth. The standard deviation remained nearly unchanged for all datasets, regardless of the reduction parameter used and the degree of variation in the bottom surface.

Considering the comparative analyzes, it is undoubted that the reduction of bathymetric data influences the shape of the digital bottom model. The shape of the resulting bottom model depends on the principle of operation of the applied reduction algorithm and the selection of its parameter value (if available). The numerical bottom models created from the reduced datasets showed insignificant differences in shape with respect to the digital bottom models obtained from the raw datasets. Notably, the datasets were found to have been significantly reduced, and the shape of the bottom was not deformed. The resulting models provided the most relevant information necessary for navigation safety. Various bottom surface shapes and different distributions of bathymetric points were used during the study. In addition to the theoretical data, which were computer-generated, real data collected on Klodno Lake were also used. The results obtained with the simulated data were confirmed by tests on real data.

Our new system, apart from the possibility of integrating data from various sensors, thereby creating a multi-dimensional and multi-temporal database about a given coastal zone, will allow visualizing these data in the form of a spatial map. Attention should be paid to the problem of the heterogeneity of these systems and the large amounts of data, which are significant research challenges. Therefore, the problem of bathymetric data reduction has been thoroughly studied. The main goal of the authors and the entire innovative project was to create a new system and new methods. All these methods, not only those related to reduction, will be able to be implemented in their own software—along with access to the source code. Once we have created our own method of reduction in our system, we will compare it with the method chosen in this publication. Artificial intelligence methods, including deep learning will be used. Looking forward, an innovative approach would present the data in the form of a spherical map, which is the future map of the new generation. This will allow for an easy and quick analysis of the situation in each coastal zone. All sensors and their system components will be placed on one vessel. Data will be collected during one route and then integrated as one. The benefits of implementing the effects of the work may include monitoring of the coastal zone (harbor quays, breakwaters, piers, locks, or marinas), inventory of the condition of navigational markings, waterways, shoreline, movements of bottom rubble, inspections of the quayside, checking the capacity of the fairway, and updating of flood hazard maps. This system will allow the checking of how the measured and visualized changes affect the surrounding environment.

## Figures and Tables

**Table 1:** Statistical summary of the test datasets.

	Dataset No. 1	Dataset No. 2	Dataset No. 3
Number of points:	1,000,000	1,000,000	1,000,000
Mean [m]:	−5.52	−5.07	−5.43
Standard deviation [m]:	1.65	2.28	1.53
Median [m]:	−5.36	−5.01	−5.57
Mode [m]:	−5.30	−2.28	−6.00
Range [m]:	7.32	9.74	9.47
Maximum depth [m]:	−9.20	−10.00	−9.61
Minimum depth [m]:	−1.88	−0.26	−0.14
Coefficient of skewness:	−0.04	−0.07	0.36
Kurtosis:	2.16	1.89	3.05

**Table 2:** Summary of the statistical measures of the No.1 raw and reduced test datasets, according to the reduction parameters used in ArcGIS Pro and HYPACK.

		ArcGIS Pro			HYPACK		
	Raw	0.05 m	0.10 m	0.20 m	0.05 m	0.10 m	0.20 m
Number of points:	1,000,000	57,647	15,306	3780	48,500	13,283	3389
Dataset size:	100.00%	5.76%	1.53%	0.38%	4.85%	1.33%	0.34%
Mean [m]:	−5.52	−5.52	−5.51	−5.50	−5.53	−5.53	−5.52
Standard deviation [m]:	1.65	1.66	1.66	1.66	1.64	1.64	1.64
Median [m]:	−5.36	−5.36	−5.35	−5.36	−5.41	−5.42	−5.40
Range [m]:	7.32	7.32	7.31	7.28	7.32	7.30	7.28
Minimum depth [m]:	−1.88	−1.88	−1.88	−1.88	−1.88	−1.88	−1.88
Maximum depth [m]:	−9.20	−9.20	−9.19	−9.16	−9.20	−9.18	−9.16

**Table 3:** Summary of the statistical measures of the No.2 raw and reduced test datasets, according to the reduction parameters used in ArcGIS Pro and HYPACK.

		ArcGIS Pro			HYPACK		
	Raw	0.05 m	0.10 m	0.20 m	0.05 m	0.10 m	0.20 m
Number of points:	1,000,000	57,790	15,427	3839	80,727	23,174	6188
Dataset size:	100.00%	5.78%	1.54%	0.38%	8.07%	2.32%	0.62%
Mean [m]:	−5.07	−5.05	−5.03	−5.00	−5.08	−5.07	−5.04
Standard deviation [m]:	2.28	2.29	2.28	2.28	2.26	2.26	2.26
Median [m]:	−5.01	−4.98	−4.96	−4.93	−5.07	−5.06	−5.03
Range [m]:	9.74	9.73	9.71	9.72	9.74	9.72	9.69
Minimum depth [m]:	−0.26	−0.26	−0.26	−0.26	−0.26	−0.26	−0.26
Maximum depth [m]:	−10.00	−10.00	−9.98	−9.98	−10.00	−9.98	−9.95

**Table 4:** Summary of the statistical measures of the No.3 raw and reduced test datasets, according to the reduction parameters used in ArcGIS Pro and HYPACK.

		ArcGIS Pro			HYPACK		
	Raw	0.05 m	0.10 m	0.20 m	0.05 m	0.10 m	0.20 m
Number of points:	1,000,000	57,286	15,014	3621	121,960	36,019	9346
Dataset size:	100.00%	5.73%	1.50%	0.36%	12.20%	3.60%	0.93%
Mean [m]:	−5.43	−5.41	−5.39	−5.35	−5.39	−5.39	−5.38
Standard deviation [m]:	1.53	1.53	1.53	1.53	1.51	1.51	1.51
Median [m]:	−5.57	−5.54	−5.52	−5.47	−5.50	−5.49	−5.48
Range [m]:	9.47	9.44	9.41	9.31	9.45	9.46	9.41
Minimum depth [m]:	−0.14	−0.14	−0.14	−0.14	−0.14	−0.14	−0.14
Maximum depth [m]:	−9.61	−9.58	−9.55	−9.46	−9.59	−9.60	−9.55

**Table 5:** Summary of the statistical measures of the raw and reduced test datasets, according to the reduction parameters used in ArcGIS Pro and HYPACK.

		ArcGIS Pro			HYPACK		
	Raw	0.05 m	0.10 m	0.20 m	0.05 m	0.10 m	0.20 m
Number of points:	13,614,483	3,349,293	1,752,338	510,658	2,934,270	1,027,925	290,556
Dataset size:	100.00%	24.60%	12.87%	3.75%	21.55%	7.55%	2.13%
Mean [m]:	−9.66	−9.99	−9.99	−10.05	−9.98	−10.07	−10.05
Standard deviation [m]:	2.46	2.59	2.67	2.68	2,60	2.68	2.70
Range [m]:	14.94	14.94	14.88	14.86	14.94	14.89	14.91
Minimum depth [m]:	−1.83	−1.83	−1.83	−1.83	−1.83	−1.83	−1.83
Maximum depth [m]:	−16.77	−16.77	−16.71	−16.69	−16.76	−16.72	−16.74

**Table 6:** Summary of the statistical measures of the raw and reduced test datasets in ArcGIS Pro software with a reduction parameter of 0.10 m.

	Raw dataset	ArcGIS Pro (0.10 m)
Number of points:	13,614,483	1,752,338
Dataset size:	100.00%	12.87%
Mean [m]:	−9.66	−9.99
Standard deviation [m]:	2.46	2.67
Range [m]:	14.94	14.88
Minimum depth [m]:	−1.83	−1.83
Maximum depth [m]:	−16.77	−16.71

## Data Availability

Not applicable.
